# Shared imaging markers of fatigue across multiple sclerosis, aquaporin-4 antibody neuromyelitis optica spectrum disorder and MOG antibody disease

**DOI:** 10.1093/braincomms/fcad107

**Published:** 2023-04-04

**Authors:** Valentina Camera, Romina Mariano, Silvia Messina, Ricarda Menke, Ludovica Griffanti, Matthew Craner, Maria I Leite, Massimiliano Calabrese, Stefano Meletti, Ruth Geraldes, Jacqueline A Palace

**Affiliations:** Nuffield Department of Clinical Neurosciences, University of Oxford, OX3 9DU Oxford, UK; Department of Neuroscience, Biomedicine and Movement Sciences, University of Verona, 37124 Verona, Italy; Department of Biomedical, Metabolic and Neurosciences, University of Modena and Reggio Emilia, 41125 Modena, Italy; Nuffield Department of Clinical Neurosciences, University of Oxford, OX3 9DU Oxford, UK; Nuffield Department of Clinical Neurosciences, University of Oxford, OX3 9DU Oxford, UK; Wexham Park Hospital, Frimley Health Foundation Trust, SL2 4HL Slough, UK; Nuffield Department of Clinical Neurosciences, University of Oxford, OX3 9DU Oxford, UK; Wellcome Centre for Integrative Neuroimaging, Oxford Centre for Functional MRI of the Brain, Nuffield Department of Clinical Neurosciences, University of Oxford, OX3 9DU Oxford, UK; Wellcome Centre for Integrative Neuroimaging, Oxford Centre for Functional MRI of the Brain, Nuffield Department of Clinical Neurosciences, University of Oxford, OX3 9DU Oxford, UK; Department of Psychiatry, University of Oxford, OX3 7JX Oxford, UK; Nuffield Department of Clinical Neurosciences, University of Oxford, OX3 9DU Oxford, UK; Wexham Park Hospital, Frimley Health Foundation Trust, SL2 4HL Slough, UK; Nuffield Department of Clinical Neurosciences, University of Oxford, OX3 9DU Oxford, UK; Department of Neuroscience, Biomedicine and Movement Sciences, University of Verona, 37124 Verona, Italy; Department of Biomedical, Metabolic and Neurosciences, University of Modena and Reggio Emilia, 41125 Modena, Italy; Nuffield Department of Clinical Neurosciences, University of Oxford, OX3 9DU Oxford, UK; Wexham Park Hospital, Frimley Health Foundation Trust, SL2 4HL Slough, UK; Nuffield Department of Clinical Neurosciences, University of Oxford, OX3 9DU Oxford, UK

**Keywords:** fatigue, multiple sclerosis, MOG-antibody disease, aquaporin-4 neuromyelitis optica spectrum disorders, resting-state functional MRI

## Abstract

Fatigue is frequently reported by patients with multiple sclerosis, aquaporin-4-antibody neuromyelitis optica spectrum disorder and myelin-oligodendrocyte-glycoprotein antibody disease; thus they could share a similar pathophysiological mechanism. In this cross-sectional cohort study, we assessed the association of fatigue with resting-state functional MRI, diffusion and structural imaging measures across these three disorders. Sixteen patients with multiple sclerosis, 17 with aquaporin-4-antibody neuromyelitis optica spectrum disorder and 17 with myelin-oligodendrocyte-glycoprotein antibody disease assessed, outside of relapses, at the Oxford Neuromyelitis Optica Service underwent Modified Fatigue Impact Scale, Hospital Anxiety and Depression Scale and Expanded Disability Status Scale scoring. A 3T brain and spinal cord MRI was used to derive cortical, deep grey and white matter volumetrics, lesions volume, fractional anisotropy, brain functional connectivity metrics, cervical spinal cord cross-sectional area, spinal cord magnetic transfer ratio and average functional connectivity between the ventral and the dorsal horns of the cervical cord. Linear relationships between MRI measures and total-, cognitive- and physical-fatigue scores were assessed. All analyses were adjusted for correlated clinical regressors. No significant differences in baseline clinical characteristics, fatigue, depression and anxiety questionnaires and disability measures were seen across the three diseases, except for older age in patients with aquaporin-4-antibody neuromyelitis optica spectrum disorder (*P* = 0.0005). In the total cohort, median total-fatigue score was 35.5 (range 3–72), and 42% of patients were clinically fatigued. A positive correlation existed between the total-fatigue score and functional connectivity of the executive/fronto-temporal network in the in left middle temporal gyrus (*P* = 0.033) and between the physical-fatigue score and functional connectivity of the sensory-motor network (*P* = 0.032) in both pre- and post-central gyri. A negative relationship was found between the total-fatigue score and functional connectivity of the salience network (*P* = 0.023) and of the left fronto-parietal network (*P* = 0.026) in the right supramarginal gyrus and left superior parietal lobe. No clear relationship between fatigue subscores and the average functional connectivity of the spinal cord was found. Cognitive-fatigue scores were positively associated with white matter lesion volume (*P* = 0.018) and negatively associated with white matter fractional anisotropy (*P* = 0.032). Structural, diffusion and functional connectivity alterations were not influenced by the disease group. Functional and structural imaging metrics associated with fatigue relate to brain rather than spinal cord abnormalities. Salience and sensory-motor networks alterations in relation to fatigue might indicate a disconnection between the perception of the interior body state and activity and the actual behavioural responses and performances (reversible or irreversible). Future research should focus on functional rehabilitative strategies.

## Teaser

Fatigue-related structural and functional MRI measures are in common across multiple sclerosis, myelin oligodendrocyte glycoprotein antibody disease and aquaporin4-antibody neuromyelitis optica spectrum disorder. Salience network alterations are correlated to global fatigue while sensory-motor network alterations to physical fatigue. Cervical cord functional connectivity disturbances appeared associated to global and physical fatigue.

## Graphical Abstract

**Graphical Abstract:**
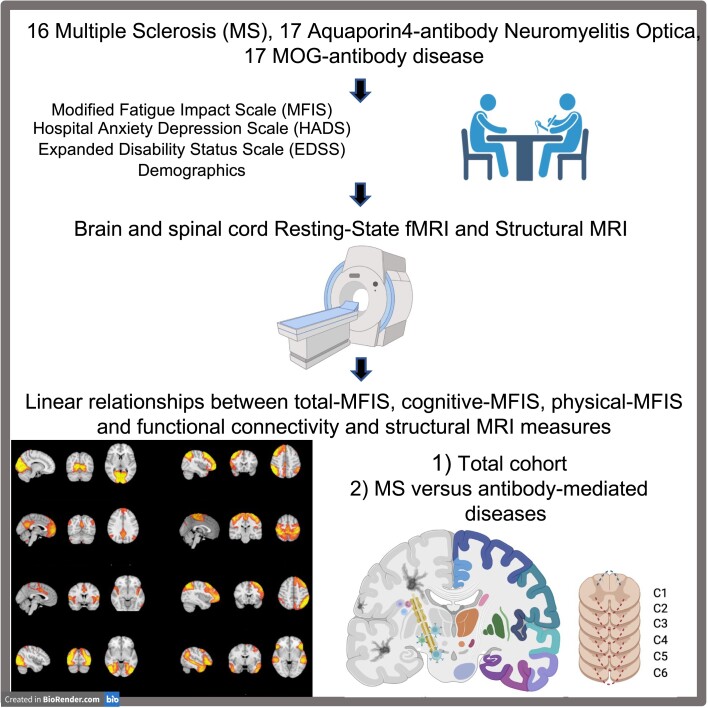

## Introduction

Fatigue is defined as a significant subjective sensation of weariness, increasing sense of effort, mismatch between effort expended and actual performance, or exhaustion independent from medications, chronic pain, physical deconditioning, anaemia, respiratory dysfunction, depression, and sleep disorders.^1,2^ Perception of fatigue is common in multiple sclerosis (MS), in neuromyelitis optica spectrum disorders associated with aquaporin-4 IgG (AQP4-NMOSD) and in myelin-oligodendrocyte-glycoprotein (MOG) antibody disease (MOGAD) patients, with a prevalence varying from 29.5% to 90%.^2,3^ However, it is unclear whether a common pathophysiological mechanism underlies fatigue in these different CNS disorders. Fatigue in MS is one of the most described symptoms,^3–5^ with up to 75% of patients reporting it during their disease course^6^ and with some considering it the most debilitating symptom independent of motor disability.^7^ Due to its disabling effect, fatigue is currently one of the top research priorities for the MS scientific society. In NMOSD, fatigue is as equally prevalent, disabling, associated with depression and pain^8–10^ and with a similar impact on quality of life^11^ as in MS.^10,12–14^ Based on non-conventional MRI study results, several hypotheses about the pathophysiological mechanisms of fatigue in MS have been proposed so far, including structural damage of white matter (WM), structural damage of grey matter (GM) and maladaptive network alterations due to distributed lesions or inflammation.^15^ On the other hand, advanced MRI measures related to fatigue have not been tested in MOGAD and AQP4-NMOSD.^2^ These considerations raise the question on whether there is a generic biomarker of fatigue across MS, MOGAD and AQP4-NMOSD, which might be useful to guide future fatigue management strategies in autoimmune demyelinating diseases of the CNS.

The primary aim of this cross-sectional study was to assess the presence of a common brain resting-state functional network biomarker of fatigue across MS, AQP4-NMOSD and MOGAD outside of relapse. Second, we evaluated the relationships between brain diffusion and structural quantitative measures and spinal cord resting-state functional, diffusion and structural quantitative measures with perception of fatigue across MS, AQP4-NMOSD and MOGAD patients.

## Materials and methods

### Population

Fifty patients at least 6 months outside of relapse (16 MS, 17 AQP4-NMOSD and 17 MOGAD) who participated in two previously published non-conventional brain and spinal cord MRI studies^16,17^ and who had additional resting-state functional imaging and matched fatigue scores were included. All subjects signed the informed consent to participate to the study approved by the Research Ethics Committee of Cambridge South (REC 17/EE/0246).

Data collection occurred from January 2018 to August 2019. The recruited MS patients fulfilled the 2017 diagnostic criteria.^18^ Seropositivity for AQP4 and MOG antibodies was confirmed using live cell-based assays (CBA).^19,20^ All AQP4-NMOSD fulfilled the NMOSD diagnostic criteria,^21^ and all MOGAD were definite positive (MOG-IgG1 ≥1:200 on live-CBA assays) and had a consistent clinical picture with MOGAD. The inclusion criteria were: (i) adult-onset of disease; (ii) presence of a 3T brain and spinal cord MRI scan performed at the Oxford Centre for Functional Magnetic Resonance Imaging of the Brain (FMRIB) at least 6 months after an acute attack and including functional imaging sequences; and (iii) performance of Modified Fatigue Impact Scale (MFIS),^22^ Hospital Anxiety and Depression Scale (HADS)^23^ questionnaires and expanding disability status scale (EDSS) scorings in the same study day as the MRI scan. We excluded isolated optic neuritis phenotypes.

#### Baseline data and clinical assessment

Clinical data collected included sex, age, self-identified race, date of disease onset, clinical phenotype at the time of MRI, as well as the number of relapses occurring before the MRI scan date from the prospective Oxford NMOSD and MS databases and the patients’ notes.

#### Fatigue questionnaire

Perception of fatigue was assessed using the MFIS questionnaire.^22^ This questionnaire consists of 21 questions, which are rated from 0 to 4 providing a total MFIS (t-MFIS) sum score ranging from 0 to 84 with nine questions assessing physical fatigue (p-MFIS), 10 questions assessing cognitive fatigue (c-MFIS) and two questions assessing psychosocial fatigue. We used the MFIS scores as continuous variables in the MRI analysis.

#### MRI data acquisition

All brain and the spinal cord MRI scans were acquired using a 3-tesla Siemens MAGNETOM® Prisma scanner at the Oxford Centre for FMRIB. The scanner was fitted with a 64-channel head and neck coil and 8-channel spine coil.

Brain MRI acquisition protocol included 3D T1 magnetization-prepared rapid gradient-echo, 3D fluid attenuated inversion recovery, diffusion spin-echo echo-planar imaging (EPI) sequences, and double inversion recovery (DIR), simultaneous multi-slice (multiband) acceleration gradient-echo echo-planar imaging (GE-EPI). The acquisition parameters of the first four sequences are described by Messina *et al*. 2021.^17^

Spinal cord MRI acquisition protocol included cervical cord 3D T1 MPRAGE; (3D)-T2 sampling perfection with application-optimized contrast using different flip-angle evolutions (3D T2 SPACE); 2D T2* weighted spoiled gradient-echo multi echo data image combination (2D T2* MEDIC); and magnetization transfer (MT) on/off and thoracic cord 3D T2 SPACE. Structural and quantitative sequences acquisition parameters are reported by Mariano *et al.*^16^ Despite all the recruited patients (*n* = 50) had brain MRI, not all the patients managed to have spinal cord MRI at the same timepoint (*n* = 45/50).

Brain and spinal cord resting-state functional MRI (RS-fMRI) sequences acquisition protocols are described in Appendix.

### Resting state functional MRI data analysis

Brain RS-fMRI data were analysed using FMRIB Software Library (FSL) 6.0.^24^ A B0 fieldmap was derived using Topup FSL tool^25,26^ calculating the difference in magnetic field distortions between two B0 spin-echo EPI acquisitions with opposite phase encoding directions. Using single-patient 3D brain-extracted T1 bias-corrected images (structural image), B0 fieldmap (radians per second) and RS-fMRI image, the pre-processing steps performed were: (i) motion correction, 100 s FWHM high pass temporal filtering, spatial smoothing, single-subject independent component analysis (ICA) using Multivariate Exploratory Linear Optimized Decomposition into Independent Components (MELODIC) 3.0 FSL tool obtaining a set RS independent components (ICs), representing noise and blood oxygenation level-dependent signal components; (ii) noise ICs were regressed out from each subject data (data denoising) using the semi-automatic tool FIX (FMRIB's ICA-based Xnoiseifier);^27,28^ and (iii) the single-subject pre-processed cleaned RS-fMRI data were registered in MNI152 standard space using FLIRT (FMRIB's linear image registration tool)/FNIRT (FMRIB's non-linear image registration tool) tools in a two-stage registration via the T1 image. From the resulting images, group-level RS networks (RSNs) (a set of spatial maps and time courses reflecting group average) were obtained by applying group-ICA with MELODIC FSL tool and a pre-defined dimensionality of 15 components. Dual regression was used to derive subject-level time series and spatial maps for each component. The resulting spatial maps entered subsequent statistical analyses.

Spinal cord functional data were processed with tools from Spinal Cord Toolbox (SCT) v4.0^29^ and FSL 6.0.^24^ First, slice-timing correction was done using FSL slice-timer, and slice-wise motion correction was performed using FSL FLIRT.^24^ Spatial smoothing (2×2×8mm^3^ full width at half maximum Gaussian kernel) was performed using the SCT tool sct_maths. FMRI Expert Analysis Tool (FEAT) was used^30^ to carry out physiological noise regression and high pass filtering (using a cut off of 100 s). Physiodata was processed using popp and pnm_evs (part of physiological noise modelling (PNM) tool in FSL). The model included cardiac, respiratory and interaction effects leading to a total of 32 regressors, as described previously.^31^ X and Y motion regressors were extracted using MATLAB R2019_a. A CSF mask was generated manually for each subject using FSLeyes to extract average time courses from CSF. The FSL FEAT was then used to regress out motion (X and Y), physiological regressors and CSF. High pass temporal filtering (100 s) was applied. The final output generated a 4D file that was used for further analysis. Anatomical data used in this analysis was segmented and registered to the PAM-50 template, as previously described. Spinal cord segmentation in the native fMRI space was done using SCT DeepSeg.^32^ Co-registration of the fMRI data to the anatomical data and template was done using the SCT tool sct_register_multimodal. *Z*-scores were extracted for each horn pair (ventral and dorsal) for every participant across the six cervical segmental levels.

### Structural and diffusion data analysis

Brain structural and diffusion MRI data were analysed using tools from the FMRIB Software Library (FSL) (fslanat and SIENAX) and freesurfer software^33^ as described by Messina *et al.*^17^ Specifically, we calculated normalized global brain volume mm^3^ (GBV), normalized white matter volume mm^3^ (WMV), normalized grey matter volume mm^3^ (GMV), normalized white matter lesions volumes mm^3^ (WMLV), number of white matter lesions, normalized averaged deep grey matter structures volumes mm^3^ (DGMV), normal appearing white matter fractional anisotropy (NAWM-FA), total white matter fractional anisotropy (WMFA), averaged mean cortical thickness (CT) and number of cortical lesions (CLs) on DIR sequences (MC).

Spinal cord structural and diffusion MRI data were analysed using SCT according to Mariano *et al*. 2021.^16^ Cervical cross-sectional area mm^2^ (C-CSA), thoracic cross-sectional area mm^2^ (T-CSA), cervical grey matter cross-sectional area mm^2^ (C-GM-CSA), whole spinal cord magnetization transfer ratio (MTR), white matter MTR (WM-MTR) and grey matter MTR (GM-MTR) scores were recorded. All brain and spinal cord quantitative values were reported as continuous variables.

### Statistical analysis

Clinical and MRI categorical variables were presented as absolute number and percentages, continuous variables as means ± standard deviation (SD) or as median and range according to whether were normally or non-normally distributed (Shapiro–Wilk normality test). Group comparisons were performed with Fisher's exact test for proportions, one way ANOVA for means and Kruskal–Wallis test for medians. Pearson's correlation coefficients were calculated across all the continuous normally distributed clinical data to identify the variables significantly associated with the t-MFIS, c-MFIS and p-MFIS scores to use as covariates or confounders of non-interest in the MRI analysis.

### Primary analysis

The primary analysis was to identify which brain RS-fMRI networks (dependent variable) were associated with t-MFIS (independent variable) and the subgroups c-MFIS and p-MFIS of the t-MFIS, and to assess whether the disease group categorisation, MS or antibody-positive diseases, altered this association adjusting for clinical confounders of non-interest. General linear models (GLM) with permutation testing (randomized) were used to find the significant linear relationship between the brain RS-fMRI spatial maps and the t-MFIS, p-MFIS, c-MFIS scores obtaining 1-*P* values corrected for family-wise error using threshold free cluster enhancement.^34^ The Harvard–Oxford cortical and subcortical structural atlases were used to locate the significant anatomical regions identified in the above analyses.^35–37^

### Secondary analyses

The secondary analysis included the use of multivariate linear regression models to assess the relationships between t-MFIS, p-MFIS, c-MFIS scores and the brain and spinal cord structural and diffusion MRI measures, and the spinal cord fMRI *z*-scores adjusting for the significant within-cohort correlated covariates with a stepwise regression method. In order to assess if the disease group was influencing the effects of the association, we used MS versus antibody disease as a categorical independent variable in the multiple regression analysis. These a priori statistical analyses were carried out using STATA 14 software.

All analyses were considered statistically significant when *P* < 0.05.

### Data availability

All data were collected and stored in accordance with GDPR guidelines. Its availability is dependent on specific collaboration and data sharing agreements made with the host organization. Analysis software and methods are publicly available.

## Results

### Clinical data and questionnaires

**Figure 1:**
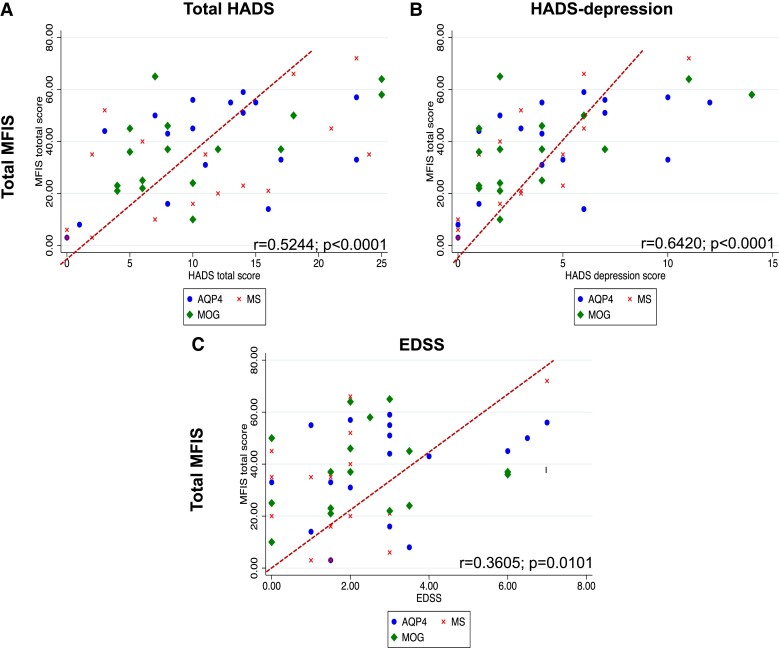
**Scatter graphs of total-MFIS scores against significant clinical variables**. According to the disease groups (cross = MS; diamond = MOG; dot = AQP4), this figure scatterplots the Pearson's correlations between t-MFIS and (**A**) HADS total score, (**B**) HADS depression score and (**C**) EDSS. MS = multiple sclerosis; MOG = myelin-oligodendrocyte-glycoprotein antibody disease; AQP4 = neuromyelitis optica spectrum disorders associated with aquaporin-4 IgG; MFIS = Modified Fatigue Impact scale; HADS = Hospital Anxiety Depression Scale; EDSS = Expanded Disability status Scale.

**Figure 2:**
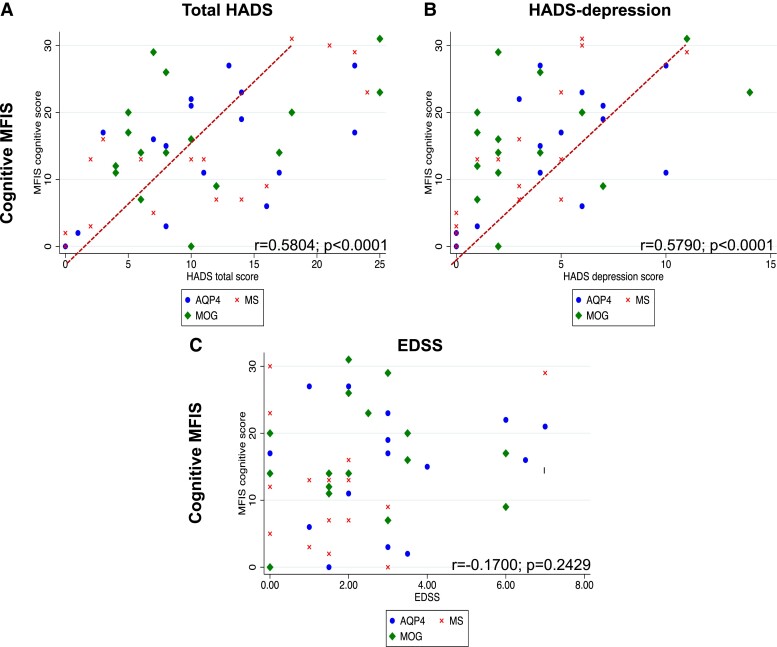
**Scatter graphs of cognitive-MFIS scores against significant clinical variables**. According to the disease groups (red cross = MS; green diamond = MOG; blue dot = AQP4), this figure scatterplots the Pearson's correlations between c-MFIS and (**A**) HADS total score, (**B**) HADS depression score and (**C**) EDSS. MS = multiple sclerosis; MOG = myelin-oligodendrocyte-glycoprotein antibody disease; AQP4 = neuromyelitis optica spectrum disorders associated with aquaporin-4 IgG; MFIS = Modified Fatigue Impact scale; HADS = Hospital Anxiety Depression Scale; EDSS = Expanded Disability status Scale.

**Figure 3:**
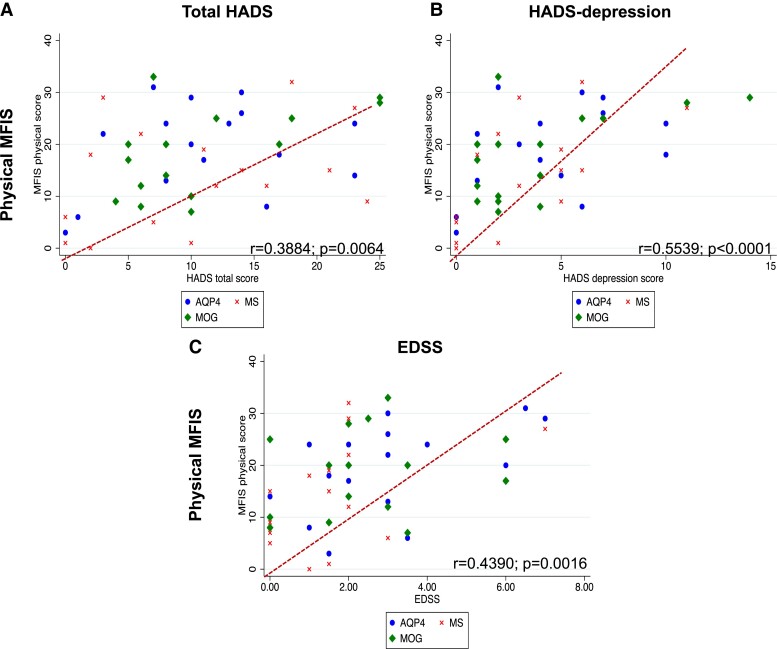
**Scatter graphs of physical-MFIS scores against significant clinical variables**. According to the disease groups (red cross = MS; green diamond = MOG; blue dot = AQP4), this figure scatterplots the Pearson's correlations between p-MFIS and (**A**) HADS total score, (**B**) HADS depression score and (**C**) EDSS. MS = multiple sclerosis; MOG = myelin-oligodendrocyte-glycoprotein antibody disease; AQP4 = neuromyelitis optica spectrum disorders associated with aquaporin-4 IgG; MFIS = Modified Fatigue Impact scale; HADS = Hospital Anxiety Depression Scale; EDSS = Expanded Disability status Scale.

Table 1 summarizes the clinical baseline characteristics of the total cohort and separately for the three disease groups. Out of the 50 patients, 62% were female with no significant difference in female-to-male ratio across the three disease groups. Mean age at the time of the assessment in the total cohort was 47.50 ± 12.40 years, with AQP4-NMOSD patients being significantly older than the other two groups (*P* = 0.0005). At the time of the study visit, total cohort median disease duration was 8.5 years (range 0–24), median EDSS was 2 (0–7) and median number of relapses before the study visit day was 2 (range 1–13); mean total cohort HADS was 10.85 ± 7.14 without significant differences across the three disease groups. Mean t-MFIS score was 35.100 ± 18.680 and, although the differences were not significant, MS patients tended to have higher c-MFIS scores, while AQP4-NMOSD and MS presented with higher p-MFIS scores, and MOGAD appeared to be slightly less affected by fatigue compared the other two groups. Supplementary Table 1 depicts the clinical variables correlated with the t-, c-, p-MFIS scores, which, if significant, were subsequently used as confounders of non-interest or covariates in the MRI analyses. Thus, we adjusted the analyses for t-MFIS using age, EDSS and HADS total score as covariates, those for c-MFIS using age and HADS total score and those for p-MFIS using age, EDSS and HADS depression sub-score. The correlations between clinical variables added in as confounders of non-interest and t-MFIS, c-MFIS, p-MFIS are depicted in Fig. 1, Fig. 2 and Fig. 3, respectively.

**Table 1:** Cohort description at the time of the MRI scan

	Total cohort *N* = 50	MS *N* = 16	MOGAD *N* = 17	AQP4-NMOSD *N* = 17	*P*-value
Sex					
Female *n* (%)	31 (62)	10	8	13	0.2220
Male *n* (%)	19 (38)	6	9	4	0.2220
Mean current age ± SD	47.5 ± 12.4	43.6 ± 6.8	42.1 ± 10.9	56.7 ± 13.4	0.0005
Race *n* (%)					
Caucasian	42 (84.0)	16 (100)	17 (100)	9 (53.0)	<0.0001
Asian	3 (6.0)	–	–	3 (17.5)	
Afro-Caribbean	3 (6.0)	–	–	3 (17.5)	
Mixed	2 (4.0)	–	–	2 (12.0)	
Mean age at onset ± SD	38.4 ± 12.4	32 ± 8.2	35.5 ± 10.9	47.2 ± 12.7	0.0005
Phenotype *n* (%)					
Isolated TM	1 (2.0)	0	1 (5.9)	0	na
ON and TM	6 (12.0)	0	6 (35.2)**	0	na
TM and BR	24 (48.0)	11 (68.9)	4 (23.5)	9 (52.9)	na
ON, TM and BR	8 (16.0)	5 (31.5)	0	3 (17.7)	na
ON, TM, BR and BS	6 (12.0)	0	6 (35.3)	0	na
Isolated BR	2 (4.0)	0	0	2 (11.8)	na
ON and BR	3 (6.0)	0	0	3 (17.7)	na
Median disease duration, years (range)	8.5 (0–24.0)	11.5 (1–19.0)	2.0 (0–24.0)	9.0 (0–24.0)	0.0774
Median number of previous relapses, (range)	2.0 (1–13.0)	4.0 (1–13)	2.0 (1–11)	2.0 (1–11.0)	0.3209
Median EDSS (range)	2.0 (0–7.0)	2.0 (0–6.0)	1.5 (0–7.0)	3.0 (0–7.0)	0.0777
MFIS					
Mean t-MFIS score ± SD	35.1 ± 18.7	37.5 ± 16.3	29.5 ± 20.8	38.4 ± 18.4	0.3214
Mean c-MFIS score ± SD	14.80 ± 8.8	16.4 ± 8.2	13.3 ± 9.7	14.8 ± 8.6	0.6015
Mean p-MFIS score ± SD	16.8 ± 9.2	17.9 ± 8.4	13.5 ± 9.9	19.3 ± 8.6	0.1689
Median of patients with t-MFIS score ≥38, *n* (%)	21.0 (42.0)	6.0 (37.5)	5 (29.4)	10.0 (58.8)	0.2190
HADS					
Mean HADS score ± SD	10.9 ± 7.1	10.6 ± 7	10.6 ± 8.2	11.4 ± 6.6	0.9413
Mean HADS depression score ± SD	4.0 ± 3.5	4.0 ± 3.8	3.3 ± 3	4.8 ± 3.6	0.4380
Mean HADS anxiety score ± SD	6.8 ± 4.7	6.6 ± 3.9	7.3 ± 5.9	6.5 ± 4.5	0.8811
Clinical MRI measures					
Median n of brain WM lesions (range)	25.0 (1.0–211.0)	63.0 (23.0–211.0)	7.0 (1.0–30.0)	20.0 (3.0–54.0)	0.0004
Median brain WM lesions volume (10^2 mm^3^) ± SD	68.3 (0.2–1082.2)	125.4 (47.94–1054)	18.2 (0.2–1082.2)	40.1 (24.7–545)	0.0004
Median *n* of cervical sc lesions (range)	0 (0–6.0)	1.0 (0–4.0)	0 (0–6.0)	0 (0–3.0)	0.2298
Mean cervical sc lesions volume (mm^3^) ± SD	98.1 ± 157.3	160.0 ± 205.0	94.8 ± 155.4	51.3 ± 95.3	0.2816

na = not applicable; this subdivision was done with only descriptive purposes and many of the variables have 0 values. MS = Multiple sclerosis; MOGAD = MOG-antibody disease; AQP4-NMOSD = aquaporin-4 antibody neuromyelitis optica spectrum disorder; SD = standard deviation; *n* = number;WM = white matter, sc = spinal cord; TM = transverse myelitis; ON = optic neuritis; BR = brain syndrome; BS = brainstem syndrome; EDSS = Expanded Disability Status Scale; t-/c-/p-MFIS = Modified Fatigue Impact Scale total/cognitive/physical scores; HADS = Hospital Anxiety and Depression Scale; BPI = Brief Pain Inventory. **asymptomatic brain lesions.

## Functional connectivity alterations related to fatigue

### Brain

**Figure 4:**
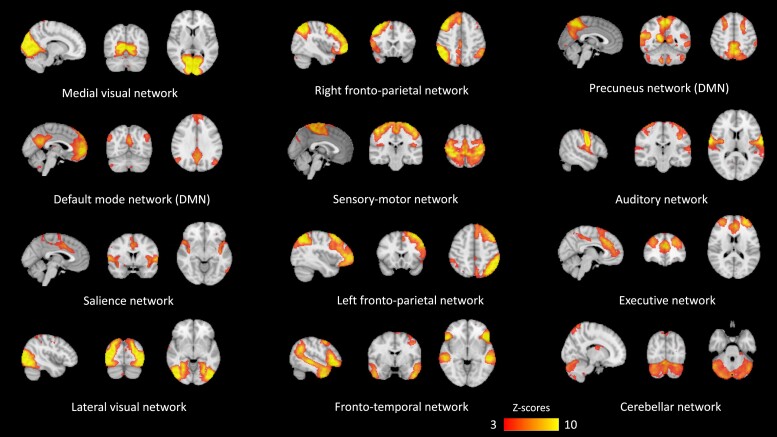
**Resting-state functional networks resulting from group independent component analysis**. Group-level RS networks (RSNs) identified after applying group-ICA with MELODIC FSL tool on 16 MS, 16 MOGAD and 16 AQP4-NMOSD patients. A threshold of *Z* = 3 has been applied here for visualisation purposes. The unthresholded maps were used as spatial templates in dual regression to derive single-subject RSN maps on the whole cohort.

A total of 12 resting-state networks (RSNs) resulted from the MELODIC group-ICA of RS-fMRI data of a group of 16 MS, 16 MOGAD and 16 AQP4-NMOSD. One MOGAD and one AQP4-NMOSD patients were excluded a priori from the group-ICA step to obtain balanced RSNs across diseases. We excluded one representative patient per group (i.e. with similar age, sex, disease duration and white matter lesion load to at least another member of the same disease group) to maintain the within-group variability during the group-ICA analysis as much as possible. The whole cohort (16 MS, 17 MOGAD and 17 AQP4-NMOSD) was then analysed in the dual regression analysis and randomized, as these statistics take care of the unbalanced groups. Figure 4 illustrates the RSNs resulting from group-ICA.

**Figure 5:**
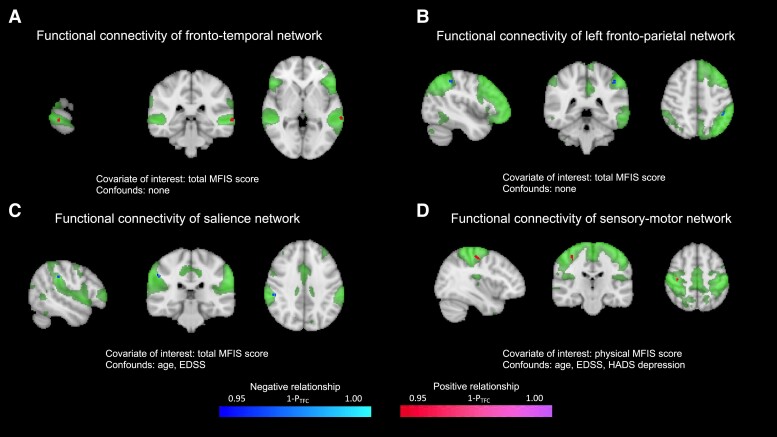
**Functional connectivity alterations within resting-state networks in relation to total and physical fatigue scores**. Significant linear relationships between the subjects’ brain RS-fMRI spatial maps and the t-MFIS (A, B and C), p-MFIS (**D**) scores obtained using general linear models (GLM) with permutation testing (randomized) and corrected for family-wise error using threshold free cluster enhancement (TFCE). Positive associations are shown in magenta-purple, negative associations are shown in blue-light blue. The corresponding resting state network from group-ICA is shown in green and thresholded at *Z* = 3 for visualization purposes. MFIS = Modified Fatigue Impact scale; HADS = Hospital Anxiety Depression Scale; EDSS = Expanded Disability status Scale.

Figure 5 illustrates the significant networks FC alterations in relation to the t-MFIS and p-MFIS scores. We found a significant positive association between the t-MFIS score and the FC of the fronto-temporal network with the left middle temporal gyrus (*P* = 0.026, Fig. 5A) and a negative relationship between the t-MFIS score and the FC of the left fronto-parietal network with the left superior parietal lobe/supramarginal gyrus (*P* = 0.026, Fig. 5B) (not adjusted for correlated clinical confounds of no-interest). Adjusting for the clinical confounds age and EDSS, we found a negative linear relationship between the t-MFIS score and the FC of the salience network with the right supramarginal gyrus of the inferior parietal lobe (*P* = 0.023, Fig. 5C). This relationship disappeared when adding the HADS total score as additional confounder; however, in an independent analysis, we did not identify any association between HADS total score and the FC of the salience network suggesting this might be a powering issue. No differences in FC of the above-mentioned networks in relation to t-MFIS were found comparing MS and antibody-mediated disease group. We did not find any alterations in the RSNs in relation to the variation of the c-MFIS scores either in the total cohort or comparing MS group to the antibody-positive diseases. On the other hand, the p-MFIS scores showed a positive linear relationship with the FC of the sensory-motor network in both the pre- and post-central gyri, even adjusting for age, EDSS and HADS depression score (*P* = 0.018, Fig. 5D). No significant group differences were detected comparing MS group to the antibody-mediated group of patients.

### Spinal cord

Forty-five out of 50 patients had a spinal cord MRI (13 MS, 16 MOGAD and 16 AQP4-NMOSD). The FC data acquired across six cervical levels (C1-C6) were averaged and plotted as two pairs: average ventral–ventral FC (V–V) and average dorsal–dorsal FC (D–D). Median *z*-score was 0.44 (range 0.140–1.070) for average V–V FC and 0.310 (range 0.070–0.630) for the average D–D FC. Average V–V FC was significantly correlated with average D–D FC (*B* = 1.00, *P* < 0.0001, *R^2^* = 0.5), but no correlations were found between the average ventral and dorsal FC and age, sex, disease duration, EDSS, C-CSA, T-CSA, GM-CSA, whole MTR, WM-MTR and GM-MTR.

In the total cohort, no linear associations were found between t-MFIS and the average V–V and D–D FC of the cervical spinal cord, even adjusting for the associated clinical regressors. These results were not affected by the disease groups (MS versus antibody-positive diseases groups). The p-MFIS was not associated with the average V–V and D–D FC of the cervical spinal cord, adjusted for clinical regressors. However, MS patients showed a trend towards a negative linear relationship between the p-MFIS and the average V–V FC of the cervical spinal cord (*B* = −27.380, *P* = 0.083, *R*^2^ = 0.720). When in addition to the other clinical confounders (age, EDSS and HADS scores), we adjusted all the functional spinal cord analyses for brain WMLV; unexpectedly, we found a negative linear relationships between both average V–V FC of cervical spinal cord and t-MFIS (*B* = −30.709, *P* = 0.021, *R*^2^ = 0.522) and p-MFIS (*B* = −27.416, *P* = 0.021, *R*^2^ = 0.548) and average D–D FC of cervical spinal cord and t-MFIS (*B* = −48.680, *P* = 0.007, *R*^2^ = 0.547) and p-MFIS (*B* = −20.768, *P* = 0.038, *R*^2^ = 0.507) (Table 3). Finally, the analyses were not influenced by the disease group (MS versus antibody-mediated diseases), and c-MFIS did not show associations with spinal cord functional measures (Table 3).

### Structural and diffusion MRI alterations related to fatigue

Table 2 illustrates the a priori correlations between structural and diffusion MRI measures and t-, c- and p-MFIS scores in the total cohort of patients.

**Table 2:** Whole cohort cross-sectional Pearson’s correlations between t-, c-, p- MFIS scores and structural and diffusion MRI measures

	t-MFIS *R* coeff	*P*-value	c-MFIS *R* coeff	*P*-value	p-MFIS *R* coeff	*P*-value
BRAIN						
Normalized global brain volume (mm^3^)	−0.0360	0.8042	0.0144	0.922	−0.0735	0.6159
Normalized WM volume (mm^3^)	−0.0396	0.7847	0.0299	0.8384	−0.1056	0.4703
Normalized GM volume (mm^3^)	−0.0257	0.8592	−0.0024	0.9868	−0.0302	0.8367
Normalized WM lesions volumes (mm^3^)	0.2021	0.1779	0.3539	0.0171	0.0971	0.5259
Number of WM lesions	0.0738	0.6260	0.1527	0.3167	0.0174	0.9094
Normalized averaged DGM volumes (mm^3^)	−0.3654	0.0091	−0.3422	0.0161	−0.2980	0.0375
Normalized averaged Thalamus vol (mm^3^)	−0.3975	0.0043	−0.3730	0.0083	−0.3189	0.0255
Normalized averaged Caudate vol (mm^3^)	−0.3186	0.0241	−0.3437	0.0156	−0.2305	0.1111
Normalized averaged Putamen vol (mm^3^)	−0.2933	0.0387	−0.2806	0.0508	−0.2333	0.1238
Normalized averaged Pallidus vol (mm^3^)	−0.2936	0.0385	−0.2815	0.0500	−0.2228	0.1238
Normalized averaged Hippocampus vol (mm^3^)	−0.3658	0.0090	−0.2872	0.0454	−0.3453	0.0151
Normalized averaged Amygdala vol (mm^3^)	−0.2206	0.1237	−0.2353	0.1036	−0.1590	0.2752
Normalized averaged Accumbens vol (mm^3^)	−0.3686	0.0084	−0.2440	0.0912	−0.4071	0.0037
Normalized brainstem vol (mm^3^)	−0.3243	0.0216	−0.2737	0.0571	−0.2812	0.0503
WM fractional anisotropy	−0.2199	0.1466	−0.2599	0.0884^a^	−0.1181	0.4452
Averaged mean cortical thickness mm^2^	−0.1315	0.3628	−0.1853	0.2025	−0.0933	0.5238
Number of cortical lesions (CLs)	0.0464	0.7620	0.1734	0.1610	−0.0511	0.7421
Spinal cord						
Cervical cross-sectional area (mm^2^)	−0.0617	0.6872	0.0038	0.9807	−0.1418	0.3587
Thoracic cross-sectional area (mm^2^)	−0.2654	0.1293	−0.1762	0.3189	−0.2918	0.0940
Cervical GM cross-sectional area (mm^2^)	−0.0881	0.5741	−0.0784	0.6216	−0.0691	0.6635
Magnetization Transfer Ratio (MTR)	−0.0266	0.8656	−0.0534	0.7369	−0.0675	0.6710
WM-MTR	−0.0667	0.6707	−0.0727	0.6472	−0.1006	0.5263
GM-MTR	0.0183	0.9075	−0.0056	0.9718	−0.0480	0.7629

a This correlation became significant when using multiple linear regression analysis adjusting for clinical covariates. MFIS = Modified Fatigue Impact scale; (t-) total score, (-c) cognitive score, (-p) physical score; *R* coeff = Pearson’s coefficient, WM = white matter, GM = grey matter, DGM = deep grey matter, CLs = cortical lesions; MTR = magnetization transfer ratio.

### Brain

Total MFIS, c-MFIS and p-MFIS scores were not associated with normalized global brain volume, adjusted for age, EDSS and HADS total score with no influence of the two disease groups.

### White matter structures

In the total cohort, c-MFIS showed a positive linear relationship with the WMLV (*B* = 0.00009, *P* = 0.018, *R^2^* = 0.430) and a negative relationship with the NAWM-FA (*B* = −67.760, *P* = 0.020, *R^2^* = 0.390) and with the WMFA (*B* = −29.160, *P* = 0.032, *R^2^* = 0.380) when adjusting for age and HADS total score. Although the mean NAWM-FA was significantly lower in the MS group compared to the antibody-disease group (*t*-test *P* = 0.05), the relationship did not change adjusting the analysis for the disease group as covariate. No significant linear relationships were found between t-MFIS, p-MFIS and normalized total WMV, normalized white matters lesions volume (WMLV), white matter lesions number (normal appearing and total), white matter fractional anisotropy (NAWM-FA and WMFA) adjusted for age, EDSS and HADS total score, and disease group (Table 3).

**Table 3:** Average functional connectivity of the ventral and dorsal horns of cervical spinal cord associated with total, cognitive and physical fatigue

t-MFIS				
Average ventral horns functional connectivity		Coeff	*P*-value	*R* ^2^
Adjusted for:	−WMLV	−34.417	0.038	0.1238
	−WMLV, age, EDSS	−40.599	0.010	0.2884
	−WMLV, age, EDSS, HADS	−30.709	0.021	0.5220
	−WMLV, age, EDSS, HADS, MS_bin	−31.233	0.023	0.5227
Average dorsal horns functional connectivity		Coeff	*P*-value	*R* ^2^
Adjusted for:	−WMLV	−48.441	0.037	0.1247
	−WMLV, age, EDSS	−57.518	0.009	0.2921
	−WMLV, age, EDSS, HADS	−48.680	0.007	0.5471
	−WMLV, age, EDSS, HADS, MS_bin	−48.620	0.008	0.5475

c-MFIS				
Average ventral horns functional connectivity		Coeff	*P*-value	*R* ^2^
Adjusted for:	−WMLV	−12.039	0.114	0.1405
	−WMLV, age	−11.881	0.123	0.1551
	−WMLV, age, HADS	−7.103	0.282	0.4093
	−WMLV, age, EDSS, HADS, MS_bin	−7.096	0.298	0.4093
Average dorsal horns functional connectivity		Coeff	*P*-value	*R* ^2^
Adjusted for:	−WMLV	−11.313	0.296	0.0259
	−WMLV, age	−17.908	0.118	0.1567
	−WMLV, age, HADS	−14.411	0.133	0.4278
	−WMLV, age, HADS, MS_bin	−14.339	0.142	0.4281

p-MFIS				
Average ventral horns functional connectivity		Coeff	*P*-value	*R* ^2^
Adjusted for:	−WMLV	−16.751	0.045	0.1054
	−WMLV, age, EDSS	−20.737	0.006	0.3706
	−WMLV, age, EDSS, HADS_D	−15.559	0.021	0.5216
	−WMLV, age, EDSS, HADS-D, MS_bin	−15.796	0.023	0.5222
Average dorsal horns functional connectivity		Coeff	*P*-value	*R* ^2^
Adjusted for:	−WMLV	−20.257	0.105	0.5403
	−WMLV, age, EDSS	−27.369	0.014	0.3397
	−WMLV, age, EDSS, HADS-D	−20.768	0.038	0.5066
	−WMLV, age, EDSS, HADS-D, MS_bin	−20.706	0.042	0.5069

t-MFIS = Modified fatigue Impact scale total score; c- MFIS = cognitive fatigue; p-MFIS = Modified fatigue Impact scale physical score; Coeff = linear regression coefficient; WMLV = white matter lesions volume; EDSS = Expanded Disability Status Scale; HADS-D = Hospital and Anxiety Scale- depression scores; MS_bin = disease groups MS versus antibody diseases.

### Grey matter structures

No significant linear relationship was found between t-MFIS, c-MFIS, p-MFIS and normalized total GMV or the averaged mean global CT even adjusting for the disease group. Normalized deep grey matter volume (DGMV) showed a significant negative linear relationship with t-MFIS when adjusted for age (*B* = −0.002, *P* = 0.011, *R^2^* = 0.140), and the disease group did not affect the result (Supplementary Fig. 1A). This relationship was particularly strong with the thalamus volume (*B* = −0.006, *P* = 0.005, *R^2^* = 0.160), the caudate nucleus volume (*B* = −0.009, *P* = 0.029, *R^2^* = 0.100), hippocampus volume (*B* = −0.012, *P* = 0.010, *R^2^* = 0.14), the nucleus accumbens volume (*B* = −0.07, *P* = 0.009, *R^2^* = 0.140) and brainstem volume (*B* = −0.002, *P* = 0.025, *R^2^* = 0.100); however, these results were no longer significant when EDSS and HADS total score were added as covariates of non-interest.

Adjusting only for age, c-MFIS score showed a negative linear relationship with DGMV (*B* = −0.0009, *P* = 0.014, *R^2^* = 0.120), especially with thalamus volume (*B* = −0.003, *P* = 0.008, *R^2^* = 0.140), and caudate volume (*B* = −0.005, *P* = 0.014, *R^2^* = 0.120), adjusting for the disease group, did not affect the results (Supplementary Fig. 1B). These significant associations were no longer significant when including the HADS total score as a covariate. On the other hand, p-MFIS, adjusted for age, showed only a trend towards a negative linear relationship with the DGMV (*B* = −0.0007, *P* = 0.054, *R^2^* = 0.090), which was not affected by the disease group (Supplementary Fig. 1C) and not significant when adjusting for EDSS and HADS depression score; mean CLs number was 3.200 ± 3.850 in MS patients, 0.320 ± 0.790 in MOGAD group. No CLs were found in the AQP4-NMOSD group. The t-MFIS, c-MFIS, p-MFIS were not associated with CLs number, even adjusting for age, EDSS, HADS scores within any of the three disease groups.

### Spinal cord

Median number of cervical spinal cord lesions in the whole cohort was 1 (range 1–6) without differences among the MS, MOGAD and AQP4-NMOSD groups (Table 1). Mean cervical spinal cord lesion volume was 260 mm^3^ ± 153 mm^3^, without significant differences across disease groups (Table 1). The t-MFIS and p-MFIS scores did not show any significant relationships with cervical spinal cord lesions volume, C-CSA, C-GM-CSA, T-CSA, whole MTR, WM-MTR and GM-MTR of the entire spinal cord, neither in the total cohort analysis nor in the diseases groups analysis (even after adjusting for correlated clinical regressors).

## Discussion

In this cross-sectional brain and spinal cord MRI study assessing generic imaging markers of fatigue across MS, MOGAD and AQP4-NMOSD, we found that (i) FC within the salience network and the left fronto-parietal network is reduced while FC within the fronto-temporal network is increased as fatigue scores increase; (ii) FC within the sensory-motor network is increased as physical fatigue scores rise; (iii) although no functional alterations were found, the increase of the cognitive fatigue scores were found to be related to the white matter lesions volumes and to the NAWM and total WM fractional anisotropy, independent of the disease type; (iv) brain structural and functional MRI abnormalities related to the fatigue, including cognitive and physical fatigue, were not influenced by the underlying demyelinating disease (MS or the antibody-mediated conditions—MOGAD and AQP4-NMOSD); and (v) spinal cord functional MRI measures were associated with the total and physical fatigue scores when the analyses were adjusted for the brain white matter lesions volume and the results were not influenced by the disease group.

Although fatigue has been highlighted to be an MS-specific symptom in CNS disease, growing evidence showed similar fatigue prevalence, depression correlation^8,12,38^ and impact on quality of life across MS and NMOSD patients.^10,12,39,40^ In the present study, for the first time, we point out that the functional and the structural MRI abnormalities found to be associated with fatigue were not influenced by the underlying diseases. In fact, our data suggest that fatigue may originate from the disruption of common brain functional networks regulating fatigue perception possibly caused by brain white matter inflammatory damage and deep grey matter demyelination and neurodegeneration. It is unlikely that other factors contributed to fatigue such as other active co-existent autoimmune disease or medications: three patients had inactive rheumatoid arthritis not requiring medication, no patients were on stimulant medication for fatigue and only six patients were on low-dose corticosteroids (*post hoc t*-test did not alter the results).

The role of global subtle inflammation on fatigue perception was recognized not only in CNS inflammatory demyelinating diseases^15^ but also in association with cancer,^41^ systemic infections,^42^ rheumatologic diseases and chronic fatigue syndrome.^43,44^ In this context, it has been suggested that either systemic or CNS inflammatory mediators could trigger CNS microglia activation leading to oxidative stress, neurotoxicity, impaired neurotransmission and alteration of the neuronal environment causing subsequently fatigue, depression, sickness behaviour and cognitive impairment,^45^ independent of the background disease causing the subtle inflammation.

In the resting-state fMRI analysis, we found that as the fatigue scores increase, the FC of the salience network with the inferior parietal lobe decreases, the FC of the left attention fronto-parietal network with the left superior parietal gyrus decreases and the FC of the fronto-temporal network with the middle temporal gyrus increases. These results lost significance when adjusting the analysis for clinical correlated confounders (age, EDSS and HADS score). Salience network findings survived to the correction for age and EDSS, but not for HADS score, suggesting a reduction in statistical power effect related to the addition of HADS score as covariate. The salience network is thought to be responsible of the interoceptive and viscero-autonomic awareness, exerting its function identifying the most homeostatically relevant among multiple internal and external stimuli (salient stimuli) that continuously approach the brain.^46^ The other key role of the salience network consists of mediating the ‘switching’ between activation of the default mode network and the executive network to guide appropriate behavioural responses to these salient stimuli.^47,48^ This network is also interconnected with the fronto-parietal network, which involves intraparietal sulcus and superior parietal lobe and mediates goal-directed, top–down attention. Interestingly, the salience network has been found to communicate with subcortical structures: visceromotor inputs informative of the current body state reach the fronto-insular cortex via vagus nerve, autonomic afferent nuclei, thalamus, the dorsal posterior insular cortex and mid-insula.^47^ Emerging evidence found that alterations within the salience network are typical of healthy controls with cognitive fatigue,^49^ many neuropsychiatric disorders,^47^ chronic fatigue syndrome in adolescents,^50^ chronic fatigue associated with cancer^51,52^ and Parkinson's disease.^53^ Moreover, recent studies on MS revealed an association between patient-reported outcomes, including fatigue, and the emotional salience network disruption.^54^ In the context of the demyelinating diseases of the CNS, we hypothesize that fatigue could emerge from any alterations (lesional or not) involving the salience network afferent and efferent pathways (including subcortical grey matter structures) and its functionally associated networks as the fronto-parietal network, which regulates the attention functions,^47^ and the fronto-temporal network, which coordinates the goal-driven behaviours.^55^ Inflammation and lesions might result therefore in a mismatch between the interoceptive and visceromotor inputs and the unexpected inability of the behavioural responses to react to these inputs.^15^ The lack of associations of fatigue scores with CLs could be because they might not be a driver of fatigue or because the majority of cortical pathology, especially in MOGAD, is not visible on 3T MRI sequences. In addition, we found that increasing physical fatigue score was associated with an increase of FC of the sensory-motor network with the pre- and post-central cortex suggesting either a dysfunction in the integration of peripheral sensory inputs to somatosensory cortex (S1) and the motor responses of the primary motor cortex (M1), as previously shown in other studies,^56,57^ or a hyperactivation of the sensory-motor network expressing the CNS compensatory mechanisms failure^58,59^ in patients with increasing levels of physical fatigue. The mismatch between perception of the needed activity and the actual performances underlying the physical fatigue might be detected via interceptive pathways and mediated by the salience network as well. Interestingly, physical fatigue appeared to associate with depression but not with anxiety scores. Perhaps, anxiety may drive the ‘fight or flight’ reaction and increase energy levels somewhat or be partly situational and temporary in the clinical research setting.

In structural MRI analyses, we found that WMLV had a strong positive effect (*R^2^* = 0.430) and NAWM-FA and total WMFA had a strong negative effect (*R^2^* = 0.390 and *R^2^* = 0.380, respectively) on cognitive fatigue adjusting for all the clinical confounders (age and HADS), while DGMV adjusted only for age showed a weaker negative effect on all the fatigue scores. The association between lower global NAWM-FA and higher fatigue scores is well-known in MS patients,^60,61^ and in our study, despite AQP4-NMOSD and MOGAD patients showed less diffusion abnormalities in the NAWM^17,62^ compared to MS patients, the association between cognitive fatigue and NAWM-FA was confirmed even adjusting for the disease group. Previous studies obtained mixed results on the association between MS-fatigue and white matter lesion load.^15,61,63–66^ Hereby, we analysed subtypes of fatigue and found only cognitive fatigue to be associated with brain WMLV and total WM and NAWM-FA independent of the disease group. In addition, many studies highlighted the role of deep grey matter, especially of the thalamus, in fatigue perception, supporting our findings and postulating that the striatal-thalamic-cortical pathway^56,67–69^ might be one of the major structures involved in fatigue pathophysiology in MS.

Finally, despite some studies suggested that in NMOSD, fatigue origin could be related to spinal cord lesions while related to brain lesions in MS,^9^ in this study, we did not find clearly structural spinal cord measures predicting global or motor fatigue. Notably, when we analysed the relationship of the average FC of the anterior and posterior horns with the fatigue scores, adjusting for the brain WMLV, age, EDSS and HADS depression scores, we noted a significant effect of the anterior horns and posterior horns FC on global and physical fatigue, although these results need to be replicated in future studies. White matter lesion load may have an independent impact on ascending and descending pathways and, subsequently, on the FC of the ventral and dorsal cervical horns in demyelinating diseases. In previous studies on healthy controls,^70^ ventral horns FC was found associated with FC of frontal motor cortices (M1), premotor cortex, dorsal premotor cortex, supplementary motor area, and anterior cingulate cortex and somatosensory partial areas (S1), whereas dorsal horns FC was found associated with somatosensory areas (S1), posterior parietal cortex, insula, thalamus, putamen, pallidum. Interestingly, all these brain areas are involved in salience network and sensory-motor network. Therefore, the reduction of FC in both ventral and dorsal horns in relation to global and physical fatigue might be explained as either a down-stream expression of the salience and sensory-motor networks dysfunctions or the origin of these networks compensatory dysregulation mechanisms.

### Limitations

The results of this study should be further validated on a larger cohort of MS, AQP4-NMOSD and MOGAD patients so that we can adjust for further potential clinical confounders and analyses can be conducted to assess if the MOGAD and AQP4-NMOSD patients cause fatigue by different mechanisms without reducing the statistical power, although we suspect it is unlikely as there is no effect of MS versus the antibody-mediated group as a whole. Nevertheless, clinical confounders as chronic pain and depression might be perceived through the salient RSN structures (i.e. insular cortex for chronic pain)^71,72^ and its interconnected networks,^73^ thus, they might represent fatigue-concomitant manifestations of salient, left fronto-parietal and fronto-temporal RSNs dysfunctions. In this study we did not include children because fMRI imaging is very sensitive to movement artefact, however, it could be interesting to validate these results on paediatric cohorts. The lack of clear associations with structural spinal cord measures supports the brain as the primary site for fatigue symptoms, however we cannot rule out acquisition challenges in spinal cord imaging hiding an effect (e.g. susceptibility and pulsation artefacts, low signal-to-noise ratio and physiological motion artifacts^74–76^). These limitations are even more pronounced in the acquisition of RS-fMRI, where susceptibility distortions and the effect of physiological noise are more pronounced than in the brain. Additionally longitudinal fMRI data comparing changes in fatigue scores during relapse would be particularly interesting. Finally, the primary sub-analyses and secondary outcomes were not individually adjusted for multiple comparisons.

## Conclusions

Although fatigue is a multidimensional symptom influenced by several different factors, it may have a similar neural pathophysiological mechanism across MS, MOGAD and AQP4-NMOSD. These mechanisms appear to affect the awareness of the body interior status mediated by the salience and fronto-parietal RSNs, and the behavioural responses to these stimuli. The high WM lesion load and the reduction of WM fractional anisotropy might decrease the connections among functionally paired cortical areas causing cognitive fatigue. The atrophy of the deep grey matter nuclei (especially thalamus) could decrease the integration between the peripheral sensory and visceromotor stimuli to the cortex, regardless of the background diseases group in global fatigue perception. The sensory-motor network was certainly affected in patients with higher physical fatigue perception, and its alteration might cause a downstream cervical spinal cord ventral and dorsal horns functional connectivity dysregulation possibly masked by brain white matter lesions volume effect on spinal cord FC. These data support findings showing that functional rehabilitative strategies such as cognitive behavioural therapy and mindfulness-based approaches could improve fatigue perception^77–82^ and functional MRI measures could be used as objective surrogate markers in future interventional studies.

## Supplementary Material

fcad107_Supplementary_Data

## Appendix

### Brain resting-state functional MRI acquisition protocol

Gradient-echo echo-planar imaging (GE-EPI) were acquired according to the UK Biobank protocol with the following parameters: resolution 2.4×2.4×2.4 mm, field-of-view (FOV) 88×88×64 matrix, repetition time (TR) 0.735 s, echo time (TE) 39 ms, GE-EPI with ×8 multi-slice acceleration, no iPAT, flip angle 52°, fat saturation. As implemented in the multiband acquisition, a separate ‘single-band reference scan’ was also acquired and used as the reference scan in head motion correction and alignment to other modalities.

### Spinal cord resting-state functional MRI acquisition protocol

Spinal cord RS-fMRI data were derived from cervical GE-EPI sequences with following acquisition parameters: resolution: 1.0 × 1.0 × 5.0 mm^3^, FOV: 128 × 128 mm^2^, TR: 1.82, TE: 30 ms, flip angle: 80, GRAPPA acceleration factor 2, PE: P≫A, Slices: 20.

## Abbreviations

AQP4-NMOSD =: neuromyelitis optica spectrum disorders associated with aquaporin-4 IgG
BOLD =: blood oxygenation level dependent
CBA =: cell-based assay
CLs =: cortical lesions
CSA =: cross-sectional area
C-CSA =: cervical cross-sectional area
CT =: cortical thickness
c-MFIS =: cognitive Modified Fatigue Impact Scale
DGMV =: normalized deep grey matter structures volume
DIR =: double inversion recovery
D–D =: average dorsal–dorsal spinal horns
DTI =: diffusion tensor imaging
DWI =: diffusion spin-echo echo-planar imaging (EPI) sequences
EDSS =: Expanded Disability Status Scale
EPI =: echo-planar imaging
FC =: functional connectivity
FEAT =: FMRI Expert Analysis Tool
FIX =: FMRIB's ICA-based Xnoiseifier
FLIRT =: FMRIB's Linear Image Registration Tool
FNIRT =: FMRIB's Non Linear Image Registration Tool
FMRIB =: Functional Magnetic Resonance Imaging Centre for the Brain
FSL =: FMRIB Software Library
FWHM =: full-width half-maximum
GBV =: global brain volume
GE-EPI =: gradient-echo echo-planar imaging
GLM =: general linear model
GM =: grey matter
GM-CSA =: grey matter cross-sectional area
GM-MTR =: grey matter magnetization transfer ratio
GMV =: normalized total grey matter volume
HADS =: Hospital Anxiety and Depression Scale
ICA =: independent component analysis
ICs =: independent components
MELODIC =: multivariate exploratory linear optimized decomposition into independent components
MFIS =: Modified Fatigue Impact Scale
MOG =: myelin-oligodendrocyte-glycoprotein
MOGAD =: myelin-oligodendrocyte-glycoprotein antibody disease
MPRAGE =: magnetization-prepared rapid gradient echo
MS =: multiple sclerosis
MT =: magnetization transfer
MTR =: magnetization transfer ratio
NAWM-FA =: normal appearing white matter fractional anisotropy
NMOSD =: neuromyelitis optica spectrum disorders
p-MFIS =: physical Modified Fatigue Impact Scale
PNM =: physiological noise modelling
RSNs =: functional resting-state networks
RS-fMRI =: resting state functional MRI
SCT =: Spinal Cord Toolbox
SD =: standard deviation
STATA =: statistical software for data science
T-CSA =: thoracic cross-sectional area
TFCE =: threshold free cluster enhancement
t-MFIS =: total Modified Fatigue Impact Scale
V–V =: average ventral–ventral cervical spinal horns
WM =: white matter
WMFA =: total white matter fractional anisotropy
WMLV =: normalized white matters lesions volume
WM-MTR =: white matter magnetization transfer ratio
WMV =: normalized total white matter volume
3DFLAIR =: 3D fluid-attenuated inversion recovery
3DT1 MPRAGE =: 3D T1 magnetization-prepared rapid gradient-echo
3D T2 SPACE =: 3D T1 sampling perfection with application optimized contrast using different flip-angle evolutions
2D T2* MEDIC =: 2D T2* weighted spoiled gradient-echo multi echo data image combination
