# Supplementary material for: Shared imaging markers of fatigue across multiple sclerosis, aquaporin-4 antibody neuromyelitis optica spectrum disorder and MOG antibody disease
Source: Brain Commun. 2023 Apr 4;5(3):fcad107. doi: 10.1093/braincomms/fcad107 (PMC10171455; doi:10.1093/braincomms/fcad107)
Supplement: fcad107_Supplementary_Data [file fcad107_supplementary_data.pdf]

Supplementary table 1: **Clinical variables correlations**

	AGE	DISEASE DURATION	EDSS	T-MFIS	C-MFIS	P-MFIS	T-HADS	D-HADS	A-HADS
AGE	1								
DISEASE DURATION	r=0.2632 p=0.0648	1							
EDSS	r=0.2049 p=0.1534	r=0.2008 p=0.1621	1						
T-MFIS	r= 0.0684 p=0.6370	r=0.0997 p=0.4907	r=0.3605 p=0.0101	1					
C-MFIS	r=0.0055 p=0.9701	r=0.1390 p=0.3407	r=-0.1700 p=0.2429	NA	1				
P-MFIS	r= 0.1121 p= 0.4433	r=0.1173 p=0.4220	r=0.4390 p=0.0016	NA	r=0.6918 p<0.0001	1			
T-HADS	r=-0.1535 p=0.2925	r=0.0775 p=0.5965	r=-0.0980 p=0.5030	r=0.5244 p<0.0001	r=0.5804 p<0.0001	r=0.3884 p=0.0064	1		
D-HADS	r=0.0195 p=0.8944	r=-0.0580 p=0.6924	r=0.1107 p=0.4488	r=0.6421 p<0.0001	r=0.5790 p<0.0001	r=0.5539 p<0.0001	NA	1	
A-HADS	r=-0.2454 p=0.0892	r=0.1592 p=0.2745	r=-0.2287 p=0.1139	r=0.3191 p=0.0254	r=0.5539 p<0.0001	r=0.2015 p=0.1696	NA	r=0.4985 p=0.0003	1

EDSS=Expanded Disability Status Scale; T-MFIS= Modified Fatigue Impact Scale total score, C-MFIS= Modified Fatigue Impact Scale cognitive subscale; P-MFIS= Modified Fatigue Impact Scale physical subscales; T-HADS= Hospital Anxiety and Depression Scale total score; D-HADS= HADS depression score; A-HADS=HADS anxiety score; r=Pearson's coefficient; p=p-value.

Legend: green box when p-value=0.1-1; yellow box when p-value=0.05-0.1; orange box when p-value=0.0001-0.05; red box when p-value<0.0001

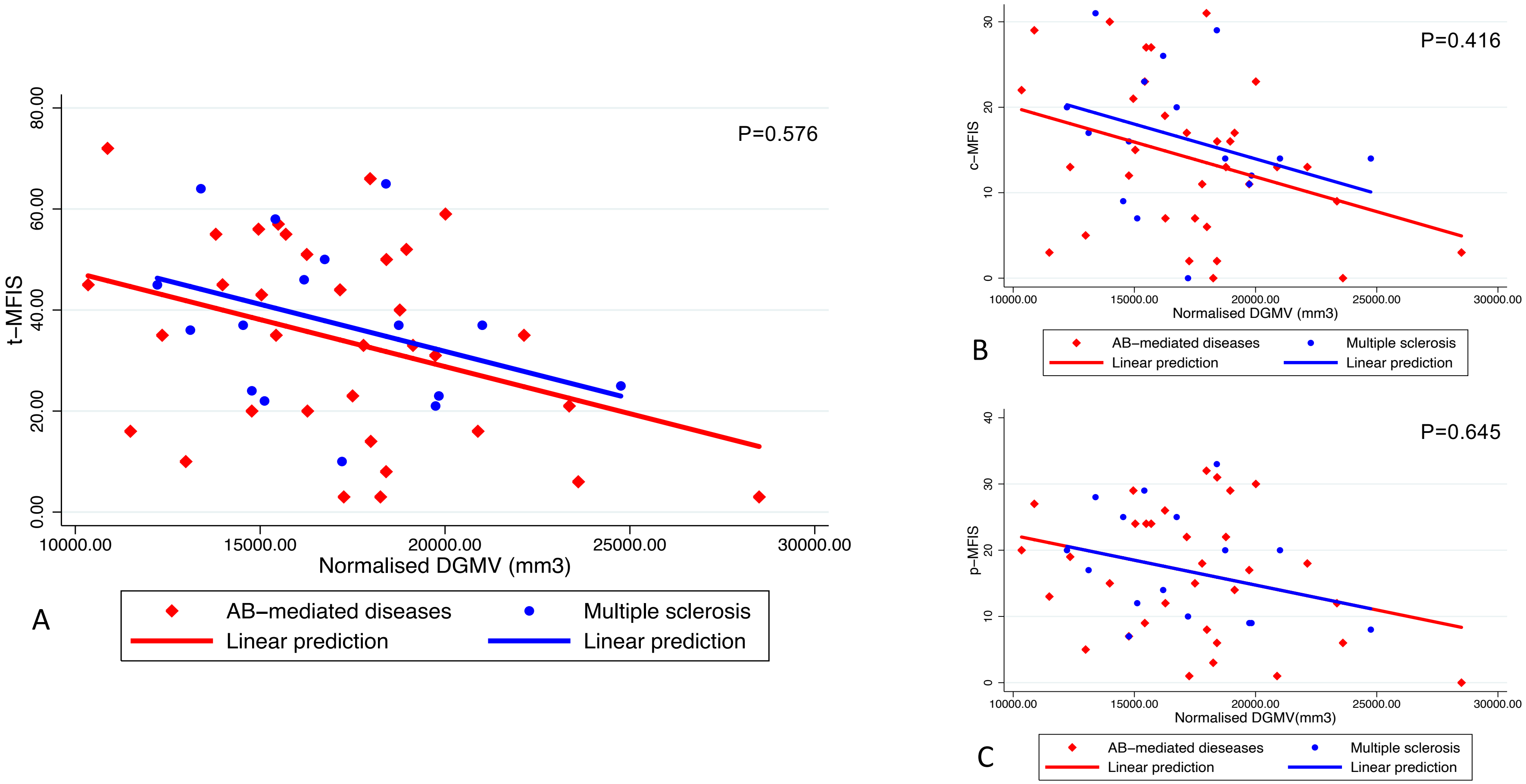

Supplementary Figure 1

Comparison of the linear relationships between MFIS scores and normalised deep grey matter volume in MS versus antibody-mediated diseases.

Graphical depiction of multiple linear regression having t-MFIS (A), c-MFIS (B), p-MFIS (C) as dependent variable and normalized deep grey matter volume as independent variable according to the disease group. No significant differences in the relationship between DGMV and fatigue scores in AB-mediated diseases and MS**.** AB-mediated diseases =antibody-mediated diseases (AQP4-NMOSD + MOGAD); DGMV= total deep grey matter volume; MFIS= Modified Fatigue Impact scale, t- (total), c- (cognitive), p-(physical)
